# Educational convergence and the equalization of happiness during the COVID-19 pandemic in China

**DOI:** 10.3389/fpubh.2026.1834105

**Published:** 2026-07-03

**Authors:** Weiwei Wang, Yan Sun, Gen Li, Yingde Tang

**Affiliations:** 1 School of Economics and Management, Jiangsu University of Science and Technology, Zhenjiang, China; 2 College of Humanities and Social Sciences, Jiangsu University of Science and Technology, Zhenjiang, China; 3 College of Earth and Environmental Sciences, Lanzhou University, Lanzhou, China

**Keywords:** China, China family panel studies, COVID-19, happiness inequality, recentered influence function

## Abstract

**Introduction:**

With the rapid development of the Chinese economy, happiness inequality is receiving increasing attention from both the scientific community and general public. The COVID-19 pandemic not only exerted a socio-economic impact but also contributed to the distribution of happiness among individuals in China. Therefore, this paper examines the impact of the COVID-19 pandemic on happiness inequality in China. We specifically analyze post-pandemic shifts in this inequality, and the urban–rural disparities.

**Methods:**

The panel data from the China family panel studies (CFPS) database (2014–2022) was applied in this study, recentered influence function (RIF) regression and Oaxaca-Blinder (OB) RIF-decomposition were performed.

**Results:**

Our results indicate that the variance of happiness declined significantly after the onset of COVID-19. The RIF regression and decomposition analyses reveal that this decline was driven by the convergence of mean happiness across educational groups—a trend already evident before the pandemic—with the happiness of the least educated rising steadily, while that of the most educated declined markedly. Once educational attainment is controlled for, the post-pandemic year coefficients attenuate substantially. Urban–rural subsample analyses confirm that this equalization pattern holds in both sectors.

**Conclusion:**

The observed reduction in happiness inequality during the pandemic reflects structural convergence along educational lines, a process that was reinforced by the narrowing of within-group dispersion for most educational groups in the early pandemic period. Whether this equalization persists beyond the zero-COVID period remains an open question for future research.

## 1 Introduction

Happiness inequality can be an important indicator of inequity within a nation. According to Veenhoven, most people dislike inequity and prefer a society with little inequality in happiness (1). Moreover, happiness inequality may add to other disparities in society. Hence, with the research on happiness becoming a hot topic in academic studies (2, 3), happiness inequality has received great attention from social science (4–6). A number of scholars have explored how best to measure the dispersion of happiness and how such inequality varies over time and/or across society (1, 7–9). With the rapid development of the Chinese economy, happiness inequality has also received increased attention from scholars (10, 11).

The COVID-19 pandemic has profoundly affected economies and societies worldwide, and China was no exception. Unlike many other countries that adopted mitigation or living-with-the-virus strategies, China implemented the world’s most stringent zero-COVID policy from early 2020 through late 2022. Alongside routine prevention and control measures, the government provided a crucial safety net for vulnerable groups through the administrative allocation of essential supplies and the rapid expansion of social assistance. This unique policy configuration reshaped the distribution of well-being among Chinese residents. While a growing body of research has examined the pandemic’s impact on mean levels of happiness, how it affected happiness inequality—the dispersion of well-being across individuals—remains poorly understood, particularly in the Chinese context. Understanding this issue not only helps assess the distributional consequences of the zero-COVID policy, but also offers lessons for other countries on how policy interventions during major public health crises can mitigate well-being inequality.

Here, using Panel data from the China family panel studies (CFPS) database (2014–2022), we attempt to answer the following questions: (1) How did happiness inequality among Chinese residents change after the onset of the COVID-19 pandemic? (2) What structural mechanisms drove these changes—in particular, what role did educational convergence play? The main contributions of this paper are as follows. First, although existing research has examined the determinants of happiness inequality in China using repeated cross-sectional data (10), evidence on how happiness inequality evolved before and after the onset of the COVID-19 pandemic remains limited. This study extends the literature by using longitudinal panel data from the China Family Panel Studies (CFPS) spanning 2014 to 2022. Second, whereas most prior research on the well-being consequences of COVID-19 has focused on mean levels of happiness, this paper shifts attention to happiness inequality and identifies the convergence of mean happiness across educational groups as the primary structural mechanism driving the observed decline.

## 2 Literature review

Research on subjective well-being and mental health has attracted considerable scholarly attention in recent decades (1–3, 12–15). The scope of subjective well-being (SWB) is broad (16), such as three main components (15). The comparability and compatibility of multiple components and measurements of SWB have also been verified previously (4, 17), and such measurements are often used interchangeably in social science research (18). Many scholars, as well as the general public and policymakers use the term ‘happiness’ as a summary description of SWB (19). The questions most frequently asked in SWB surveys are life satisfaction or happiness (16), and this self-reported measure of well-being is reasonably valid, reliable, and comparable across nations (20).

A growing body of research has examined the impact of the COVID-19 pandemic on residents’ happiness and subjective well-being (21, 22). To study happiness inequality, various measures of divergence have been developed; these include the standard deviation (SD), Gini coefficient, and ranges between lowest and highest points (or between specific points) (4). Although each indicator has advantages and shortcomings, the SD is currently the simplest and most informative, that is the summary measure that has been applied most often to measure happiness inequality (5, 7, 20, 23, 24).

Many studies have investigated the happiness inequality and its determinants in different nations, especially in developed countries. Clark et al. showed that happiness inequality has decreased in Western nations that have experienced income growth, with the extension of availability of various public goods helping to explain this greater happiness homogeneity (25). Data from Japan also recorded an overall downward trend in happiness inequality between 2003 and 2013, with RIF regressions suggesting that household income has a negative and significant effect on happiness inequality (26). However, research from Germany demonstrated and increase in happiness inequality between 1992 and 2007, with RIF regression results showing that the trends in happiness inequality were mainly driven by composition effects, education had the impact of reducing inequality, while unemployment contributed to an increase in happiness inequality (27). Stevenson & Wolfers examined how the dispersion of happiness evolved over the period 1972–2006 in the United States, they discovered a substantial fall in happiness inequality in the 1970s and 1980s, followed by a reversal and subsequent rise equating to approximately one-third of the initial decline (28). Meanwhile, Clark et al. showed that happiness inequality increased with income inequality, but decreased with income growth, which may explain the rebound in happiness inequality in Germany and the United States (29). In South Africa, there was a decrease in happiness inequality between 2008 and 2014, with income inequality having a significantly positive impact on happiness inequality (Kollamparambil), consistent with the findings of Clark (30). Ott compared 130 countries and revealed that good governance could lower the inequality of happiness among citizens (5).

For researches on China, Zhang showed that happiness inequality first increased and then decreased as per-capita gross domestic product increased in Chinese provinces (11). Yang et al. demonstrated that happiness inequality in China increased between 2009 and 2015 (10), with the results of RIF regression suggesting that an increase in income or education level has a reducing impact on happiness inequality, a decomposition analysis showed that happiness inequality in China is mainly caused by coefficient effects. The urban–rural dual structure has profoundly shaped the subjective well-being of Chinese residents. In recent years, a growing body of research has examined the evolving trends and structural drivers of the urban–rural well-being gap. Nationally representative data reveal a rising trend in subjective well-being in China over the past decade, with particularly notable improvements in rural areas (31). A study using social media data from 107 Chinese cities further documents a significant decline in subjective well-being inequality during urbanization, with SWB rising among the least happy and declining among the happiest (32). Among the various factors driving the urban–rural well-being gap, education has been identified as the most important contributor: research using the 2013–2021 Chinese Social Survey shows that education accounts for at least half of the total urban–rural well-being gap (33). Taken together, these studies provide important structural context for understanding the core finding of this paper—that the convergence of mean happiness across educational groups was the dominant force behind the equalization of happiness during the pandemic.

Since the outbreak of COVID-19, a large body of research has examined its impact on residents’ subjective well-being, focusing primarily on changes in mean levels of well-being or the psychological distress of specific groups. For example, the self-employed in the United Kingdom experienced a marked decline in subjective well-being due to sharp reductions in working hours and income (34), while children in Bangladesh suffered impaired subjective well-being as a result of family displacement, parental job loss, and food poverty (35). Evidence from China indicates that neighborhood governance played a vital role in maintaining residents’ happiness (36). In addition, high school graduates in Germany experienced a brief improvement in well-being at the onset of school closures, but their mental health deteriorated substantially as the pandemic wore on, adversely affecting their educational and career plans (37). A longitudinal study in Canada found that the pandemic exerted the largest negative effects on those with the lowest prior well-being, while post-secondary education served as a protective factor against well-being decline (38). Finally, among community-based physical activity participants in the United Kingdom, happiness and social connections declined significantly due to lockdowns, although those who were more active before the pandemic were less adversely affected (39).

However, it is relatively new to study the impact of COVID-19 on the happiness inequality of residents. Delhey et al. suggested that the unfolding pandemic did not heighten well-being inequalities in Germany and the United Kingdom (40). Araki suggested that there was a downward trend of happiness inequality in Japan during the pandemic period (2020–2022) (4). These studies mainly focused on developed countries; hence, it remains necessary to conduct research on the changes of happiness inequality in China, the largest developing country in the world.

## 3 Theoretical framework and hypotheses

The Chinese context produced a distinct distributional outcome, centered on three interrelated features. First, the government-led buffer mechanism. Unlike countries with limited social protection, China’s stringent dynamic zero-COVID policy was accompanied by a rapid expansion of social assistance and the administrative allocation of essential supplies during the policy period. This provided a crucial safety net for the most vulnerable, preventing a further deterioration of happiness at the lower end of the distribution—consistent with the broader finding that good governance can lower happiness inequality (5). Second, the experiential convergence mechanism. The dynamic zero-COVID policy disproportionately curtailed the professional and consumption activities of the highly educated, while the homogenization of daily life narrowed the experiential gap between higher- and lower-status groups. Consequently, the highly educated experienced a marked decline in happiness, while the less educated—protected by the expanded safety net and reduced social comparison pressures—saw their happiness remain stable or modestly improve. Third, education as the structural linchpin. Prior research consistently shows that education exerts a reducing effect on happiness inequality (10, 27). The pandemic-induced convergence of happiness across educational groups thus made education the central axis along which happiness equalization unfolded.

The strength of these three mechanisms may differ between urban and rural areas. The two sectors differ substantially in educational composition—lower-educated groups are disproportionately concentrated in rural areas, while the highly educated are concentrated in cities—and rural households are not economically isolated, relying on urban labor markets through migration and remittances. We therefore examine urban and rural patterns separately in the empirical analysis to test the robustness of our core findings, without imposing identical mechanisms across sectors.

Based on this framework, we derive two testable hypotheses:

*Hypothesis* 1: The overall variance of happiness declined significantly after the onset of the COVID-19 pandemic compared to the pre-pandemic period.

*Hypothesis* 2: This decline is primarily driven by the convergence of mean happiness across educational groups. Once educational attainment is controlled for, the post-pandemic year coefficients should attenuate substantially, while within-group dispersion should remain relatively stable.

## 4 Materials and methods

### 4.1 Data

This study uses data from the China Family Panel Studies (CFPS) to investigate happiness inequality in China before and after the onset of the COVID-19 pandemic. The CFPS is a nationally representative, large-scale, longitudinal social survey implemented by the Institute of Social Science Survey at Peking University. Launched in 2010, the survey has been conducted biennially thereafter, with the 2022 wave recently released. The CFPS employs a three-stage systematic probability-proportional-to-size (PPS) cluster sampling design, covering 25 provinces, municipalities, and autonomous regions that represent approximately 95% of China’s population. The 2010 baseline survey completed interviews with 14,960 households and 42,590 individuals. The CFPS aims to reflect changes in Chinese society, economy, population, education, and health, providing valuable data for both academic research and public policy decision-making. Since its release, the CFPS has been widely used to study a broad range of economic and social issues in China (41–44).

The variable of happiness was not investigated, or the survey content was inconsistent with subsequent investigations for adults prior to 2014. Owing to a large number of missing happiness values in 2016, the RIF regression performed herein only used data from 2014, 2018, 2020, and 2022 for analysis. By merging the personal and household CFPS databases, and removing data that failed to match, there were a total 25,498 individuals in 2022, 27,673 individuals in 2020, 36,735 individuals in 2018, and 31,392 individuals in 2014. However, many variables had missing values, which reduced the size of observations used in subsequent regression analysis.

### 4.2 Variables

In the CFPS project, respondents answered the question “Are you happy?” in the context of an 11-point scale, with 0 being the lowest and 10 the highest. We used this measurement of happiness to study happiness inequality herein.

Our analysis focuses on changes in happiness inequality before and after the outbreak of the COVID-19 pandemic. We use survey year as a categorical variable, with 2014 serving as the reference group and separate dummy variables for 2018, 2020, and 2022. This specification allows us to compare happiness variance across four time points—two before the pandemic (2014 and 2018) and two after its onset (2020 and 2022)—without imposing the assumption that the two pre-pandemic or the two post-pandemic years are identical.

All models control for a set of demographic and socioeconomic variables that prior research has identified as important correlates of subjective well-being. These include age, marital status, gender, and urban–rural residence. In addition, all specifications include province fixed effects to account for unobserved time-invariant regional heterogeneity, and survey year fixed effects to capture temporal shifts in happiness variance. Descriptive statistics for all variables are reported in Table 1. In subsequent models, we further introduce educational attainment dummies to examine the role of between-group convergence in driving changes in happiness variance.

**Table 1:** Variable descriptions in year 2018 and 2020.

Variables	Variable descriptions	2018 Mean (SD) /Frequency (%)	2020 Mean (SD) /Frequency (%)	2022 Mean (SD) /Frequency (%)
Happiness	Score from 0 to 10, “0” be the lowest score	7.546 (2.165)	7.547 (2.080)	7.504 (2.068)
Demographic variables				
Female	Female = 1	18,452 (50.23)	13,830 (49.98)	12,722 (49.89)
	Male = 0	18,283 (49.77)	13,841 (50.02)	12,776 (50.11)
Age	Continuous variable	44.887 (19.361)	44.311 (19.446)	44.939 (19.750)
Marriage dummy	Having a spouse in marriage = 1	23,716 (78.30)	17,220 (76.62)	15,609 (75.53)
	Else = 0	6,573 (21.70)	5,255 (23.38)	5,057 (24.47)
Education dummy	Illiterate / Semi-literate	9,366 (25.72)	6,635 (24.04)	5,346 (20.98)
	Primary school	7,844 (21.54)	5,667 (20.53)	4,987 (19.57)
	Middle school	10,031 (27.54)	7,631 (27.65)	7,245 (28.43)
	High school / vocational school	5,299 (14.55)	4,132 (14.97)	3,916 (15.37)
	Junior college / University	3,749 (10.29)	3,377 (12.24)	3,791 (14.88)
	Postgraduate	133 (0.37)	158 (0.57)	198 (0.78)
Urban resident	Urban = 1	17,120 (49.40)	13,139 (50.40)	13,095 (51.86)
	Rural = 0	17,535 (50.60)	12,931 (49.60)	12,154 (48.14)

SD, standard deviation.

### 4.3 Econometric methods

RIF regression has been widely used to study the determinants of happiness inequality at the micro-economic level (10, 26, 27). Herein, to identify the effect of the COVID-19 pandemic on happiness inequality among residents in China, empirical analysis was undertaken based on RIF regression, where we used the variance of happiness to reflect happiness inequality. We estimated the happiness inequality function as follows:


$$\mathrm{RIF}(\text{happiness}\_\mathrm{var}\text{iance})=\beta_{0}+\sum \beta_{k}x_{\mathrm{ik}}+\varepsilon_{i}$$ (1)

where *x_ik_* is the *k*-th explanatory variable of the *i*-th individual, *ε_i_* is an error term. According to (45), estimation of the RIF was calculated as below (Equation 2):


$$\mathrm{RIF}(y_{i},\sigma_{Y}^{2})=(y_{i}-\mu_{Y}^{2})$$ (2)

Where, $\sigma_{Y}^{2}$ denotes the variance of the outcome variable *Y*, $u_{Y}^{2}$ denotes the mean of outcome variable, and $y_{i}$ denote the value of the *i*-th individual of outcome variable.

Traditionally, the OB decomposition method is employed to analyze the causes of group differences, such as those in the two periods before (groups 0) and after the onset of the COVID-19 pandemic (groups 1) (46, 47), as shown in Equation 3:


$$\mathrm{\Delta v}=v(F_{1})-v(F_{0})=v_{1}-v_{0}$$ (3)

Where *v*_0_ and *v*_1_ denote dependent variable statistic of groups 0 and groups 1 respectively, Δv denotes the change in the dependent variable statistic in which we are interested; this is variance herein. $v(F_{1})$ and $v(F_{0})$ denote the statistics of two respective categories.

Consider a linear case; after the OLS regression of RIF on *X*, we obtain the following Equation 4:


$$\mathrm{\Delta v}=(v_{1}-v_{c})+(v_{c}-v_{0})=\Delta_{S}^{v}+\Delta_{X}^{v}$$ (4)

Where, the second term $\Delta_{X}^{v}$ reflects the gap attributed to differences in characteristics. The first term $\Delta_{S}^{v}$ reflects the differences attributed to relationships between *Y* and *X* (structure effect). Additionally, under the assumptions of conditional independence and overlapping support, the aggregate structure effect can be identified and interpreted as a treatment effect (45).

Linear regressions and their approximations were used to identify *v_c_*. Specifically, following Equation 1, separate RIF regressions can be estimated for each group, so that the counterfactual statistic can be identified as follows (Equations 5–7):


$$v_{1}=E[\mathrm{RIF}\{y,v(F_{Y|T=1})\}]={\bar{X}}^{1’}{\hat{\beta }}^{1}$$ (5)

$$v_{0}=E[\mathrm{RIF}\{y,v(F_{Y|T=0})\}]={\bar{X}}^{0’}{\hat{\beta }}^{0}$$ (6)

$$v_{c}={\bar{X}}^{1’}{\hat{\beta }}^{0}$$ (7)

Where ${\bar{X}}^{1\prime }$ and ${\bar{X}}^{0\prime }$ are the unconditional mean of explanatory variables of group 1 and group 0, respectively. ${\hat{\beta }}^{1}$ and ${\hat{\beta }}^{0}$ are the coefficients of RIF regression of two groups, respectively (45).

## 5 Results

### 5.1 Descriptive statistics

**Figure 1:**
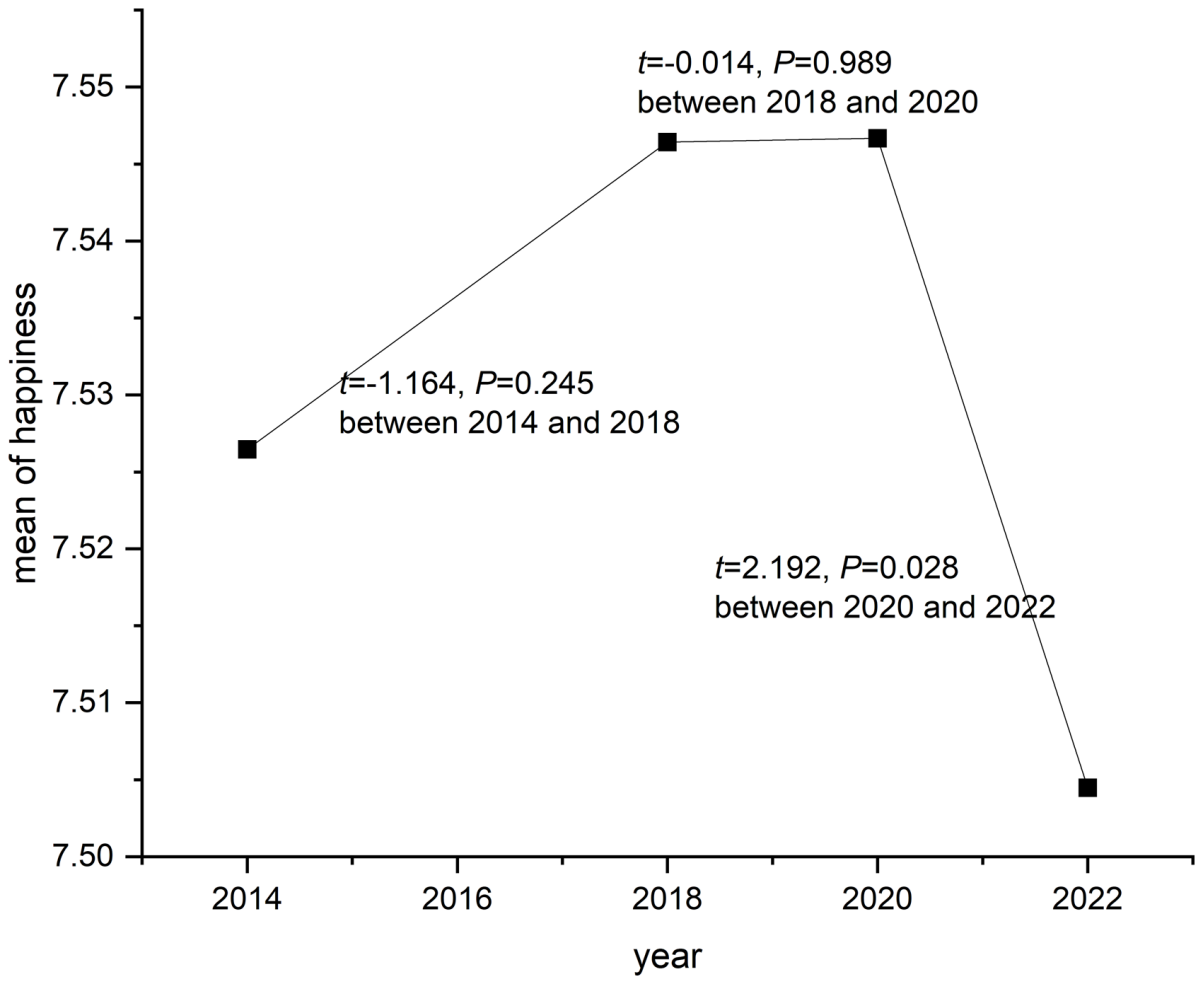
Average score of happiness in different years.

The mean of happiness from 2014 to 2022 is shown in Figure 1. From 2014 to 2020, there was no significant change in the average happiness of Chinese residents. The average happiness level was 7.526 in 2014 and 7.546 in 2018, with no significant difference between the 2 years (*t* = −1.164, *p* = 0.245). The average happiness level in 2020 was 7.547, again showing no significant change between 2018 and 2020 (*t* = −0.014, *p* = 0.989). Perhaps due to the impact of COVID-19, the average happiness of Chinese residents declined significantly between 2020 and 2022 (*t* = 2.192, *p* = 0.028), with an average decline of 0.042.

**Figure 2:**
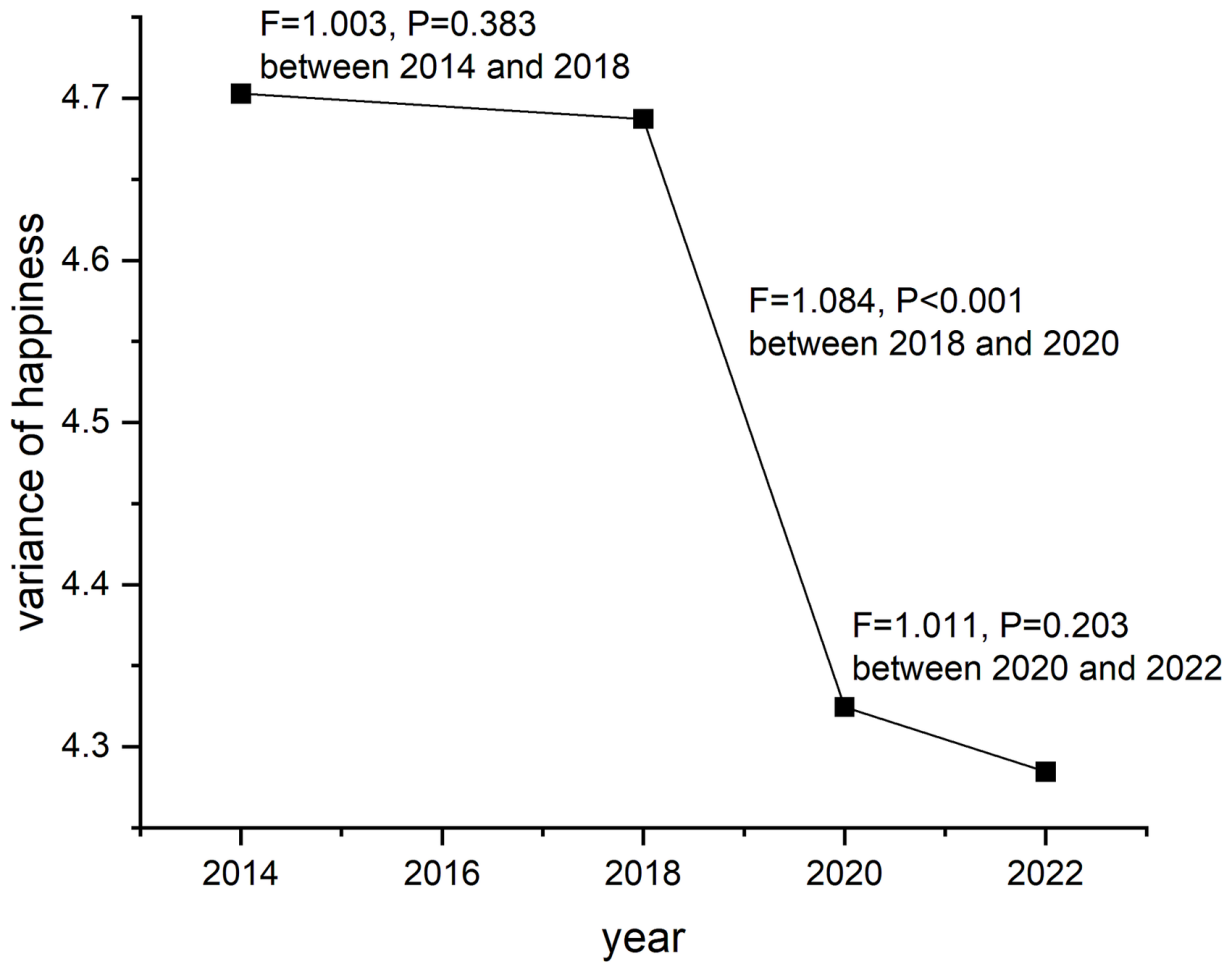
Variance differences of happiness in different years.

The variance of happiness from 2014 to 2022 is shown in Figure 2. There was no significant difference in the variance of the happiness of residents between 2014 and 2018 (*F* = 1.003, *p* = 0.383), while there was a significant decrease in the variance of happiness between 2018 and 2020 (*F* = 1.084, *p* < 0.001), coinciding with the onset of the COVID-19 pandemic. This was followed by a period of stability between 2020 and 2022, during which the variance did not change significantly. The temporal coincidence between the pandemic outbreak and the decline in variance is suggestive. Whether the reduction in happiness inequality persisted after the relaxation of the zero-COVID policy in late 2022 remains to be seen in future data.

**Figure 3:**
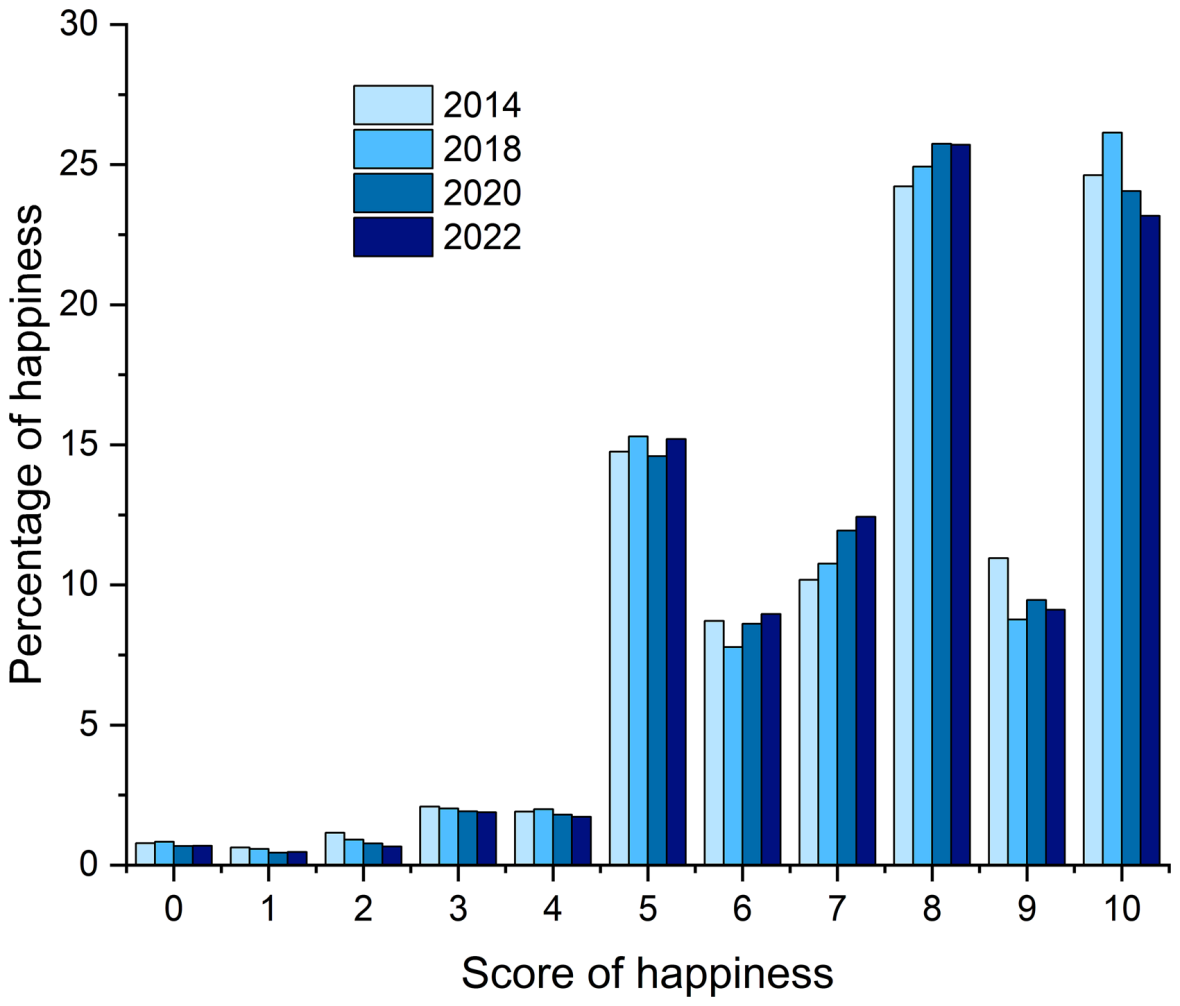
Distribution of happiness in different years.

Figure 3 present the distribution of happiness scores across survey waves, illustrating how the decline in variance occurred without a corresponding shift in the mean. Two features are noteworthy. First, the proportion of responses at both ends of the distribution declined after 2020. The share of individuals reporting the highest score (10 points) fell steadily from 2018 to 2022, while the share reporting scores of 4 and below also decreased. Second, the middle and upper-middle range became more densely populated, with the proportion of scores between 5 and 9 increasing noticeably during the pandemic period. This redistribution toward the center—fewer individuals at both extremes and more in the middle—mechanically reduced the variance while leaving the overall mean largely unchanged. In substantive terms, the equalization of happiness was driven not by a uniform shift in either direction, but by a convergence toward moderate levels of well-being.

**Figure 4:**
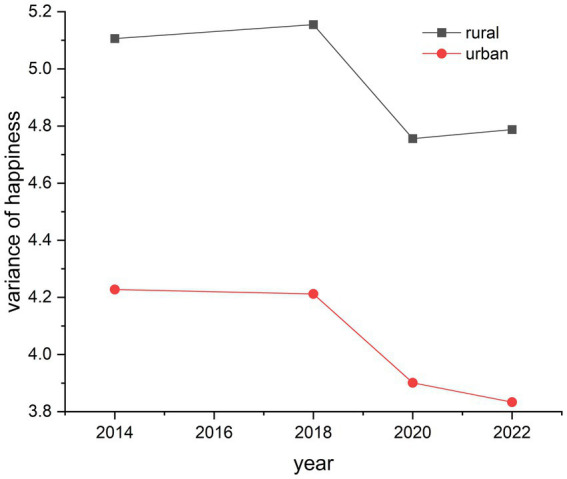
Differences in variance of happiness between urban and rural areas in different years.

**Figure 5:**
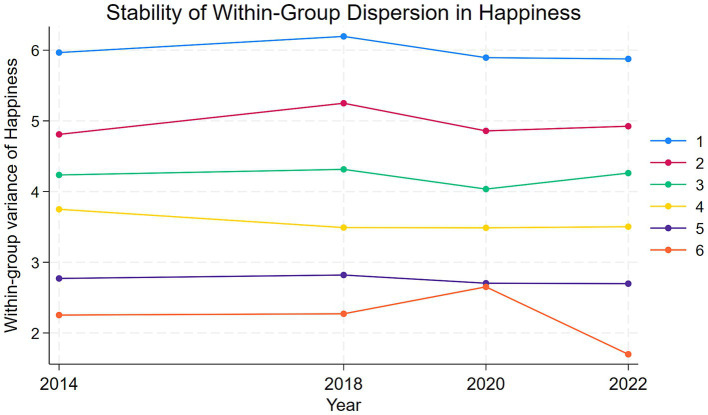
Within-group variance changes across educational groups. Note: 1 = Illiterate / Semi-literate; 2 = Primary school; 3 = Middle school; 4 = High school / vocational school; 5 = Junior college / University; 6 = Postgraduate.

Figures 4, 5 together present the trends in happiness variance by urban–rural residence and within-group dispersion by educational attainment, offering complementary perspectives on the structural sources of the observed equalization.

Across all survey waves, rural happiness variance is consistently higher than urban variance. In 2014, rural variance stood at 5.11 compared with 4.23 in urban areas; by 2022, rural variance had declined to 4.79 and urban variance to 3.83. The education composition of the two sectors helps account for this persistent gap. Taking 2022 as an illustrative year, those with primary school education or below account for 51.8% of the rural sample, compared with roughly 30.3% of the urban sample. Conversely, junior college, undergraduate and postgraduate holders make up just 7.3% of the rural population, versus 23.3% of the urban population. As Figure 5 shows, lower-educated groups exhibit substantially larger within-group variance—the illiterate and semi-literate group, for instance, has a variance above 5.8 throughout the period, while the junior college and bachelor’s degree group remains below 2.9. The disproportionate concentration of these high-variance groups in rural areas mechanically raises the overall rural variance and, to a considerable extent, explains the persistent urban–rural gap.

Two further observations qualify this structural account. First, both urban and rural areas experienced a notable decline in happiness variance in 2020—urban variance fell from 4.21 in 2018 to 3.90, and rural variance from 5.15 to 4.76. Second, after 2020, the two sectors diverged: urban variance continued to decline to 3.83 in 2022, whereas rural variance edged up slightly to 4.79. This divergence serves as a reminder that rural areas in contemporary China are not economically isolated systems. A substantial share of rural households depends on urban labor markets through migration and remittances; prolonged urban economic disruptions during the pandemic likely generated adverse spillovers that placed upward pressure on rural happiness variance, partially offsetting the equalizing effect of the expanded social safety net. The education-based account, while informative, should therefore be understood as a partial rather than complete explanation. The precise mechanisms underlying the urban–rural divergence warrant further investigation.

### 5.2 Recentered influence function regression results

Table 2 reports the estimation results of the RIF variance regression. Model 1 includes only basic demographic variables such as age, marital status, gender, urban–rural residence, and province fixed effects. Using 2014 as the reference group, the coefficient for 2018 is −0.077 and statistically insignificant (*p* = 0.185), indicating that the overall variance of happiness among Chinese residents did not undergo a statistically significant change between 2014 and 2018. However, the coefficients for 2020 and 2022 are −0.405 and −0.433, respectively, both significant at the 1% level. This result suggests that following the outbreak of the COVID-19 pandemic, the distribution of residents’ happiness exhibited a significant trend toward reduced variance. Specifically, the variance of happiness in 2022 decreased by 0.433 compared to 2014.

**Table 2:** RIF regression results of happiness variance in China.

	Model 1: Happiness, variance	Model 2: Happiness, variance
Age	0.040^***^ (0.001)	0.013^***^ (0.002)
Year dummy (Reference group: 2014)		
Year of 2018	−0.077 (0.058)	0.112^*^ (0.058)
Year of 2020	−0.405^***^ (0.061)	−0.121^**^ (0.061)
Year of 2022	−0.433^***^ (0.062)	−0.045 (0.063)
Education level	No	Yes
Marriage dummy	−0.946^***^ (0.065)	−0.903^***^ (0.066)
Female	0.166^***^ (0.043)	−0.097^**^ (0.044)
Urban	−0.687^***^ (0.046)	−0.230^***^ (0.047)
Province dummy	Yes	Yes
_cons	3.565^***^ (0.223)	6.442^***^ (0.242)
*N*	102,538	100,786
adj. *R*^2^	0.019	0.031

Robust Standard errors in parentheses. *p < 0.1, **p < 0.05, ***p < 0.01.

Model 2 further incorporates educational attainment dummies on the basis of Model 1. The coefficients for 2020 and 2022 attenuate substantially: the coefficient for 2020 rises from −0.405 to −0.121 (*p* = 0.049); the coefficient for 2022 rises from −0.433 to −0.045 and becomes statistically insignificant (*p* = 0.475). The dramatic change from −0.433 to −0.045 indicates that the observed reduction in happiness variance during the pandemic is almost entirely driven by structural changes across educational groups. Once educational attainment is held constant, the year effect largely disappears.

**Figure 6:**
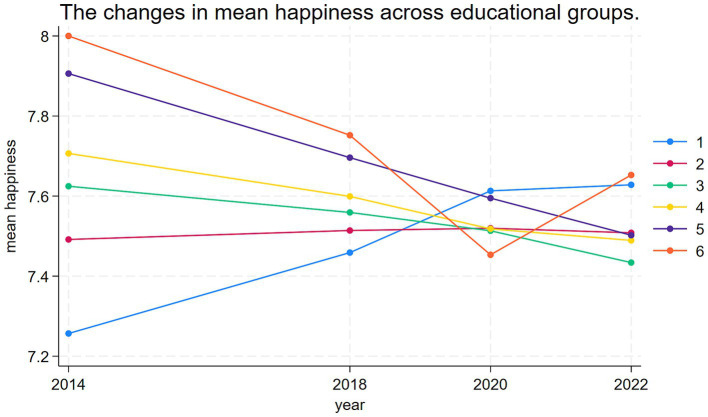
Mean happiness trends across educational groups. Note: 1 = Illiterate / Semi-literate; 2 = Primary school; 3 = Middle school; 4 = High school / vocational school; 5 = Junior college / University; 6 = Postgraduate.

Figures 5, 6 provide intuitive mechanistic evidence for the regression results reported above. In terms of between-group means, the happiness gap between the highest education group (postgraduate) and the lowest education group (illiterate/semi-illiterate) narrowed persistently from 0.743 in 2014 to 0.293 in 2018, and further to merely 0.024 in 2022, indicating near-complete convergence. This convergence was driven by the opposing movements of the two ends: the happiness of the lowest education group rose steadily, while that of the highest education groups declined markedly. Notably, the bulk of this convergence had already occurred by 2018, yet the overall happiness variance in that year did not register a statistically significant decline (*β* = −0.077, *p* = 0.185). Between 2014 and 2018, the variance-compressing effect of narrowing between-group gaps was offset by a slight widening of within-group dispersion. Once education is controlled for—thereby stripping out the between-group component—the year coefficient for 2018 turns positive (*β* = 0.112, *p* = 0.055), revealing the underlying increase in within-group variance during this period.

The pattern shifted markedly after the outbreak of the pandemic. Between 2018 and 2020, within-group dispersion narrowed for most educational groups, working in the same direction as the continued between-group convergence to drive a sharp decline in overall variance (see Figure 5). This trend largely continued into 2020–2022, during which within-group dispersion stabilized for most groups (and even contracted further among postgraduate respondents), helping to maintain overall variance at the lower level achieved in 2020. This explains why the year coefficients for 2020 and 2022 are large and significant in Model 1 (*β* = −0.405 and −0.433, respectively), but drop sharply once education is controlled for in Model 2, the 2022 coefficient becomes insignificant (*β* = −0.045, *p* = 0.475), while the 2020 coefficient remains only marginally significant (*β* = −0.121, *p* = 0.049).

These descriptive patterns, together with the RIF variance regression results, form a coherent chain of evidence. Without controlling for education, the year dummies capture the combined effect of narrowing between-group gaps and changing within-group dispersion. The pre-pandemic period (2014–2018) saw these two forces offset each other, resulting in no significant change in overall variance. Once education is controlled for, the between-group convergence is absorbed by the education dummies, and the year coefficients drop substantially. These findings are consistent with the interpretation that the observed equalization of happiness during the pandemic reflects the convergence of mean happiness across educational groups, which was already underway before the pandemic but was only fully realized in terms of overall variance reduction once the countervailing within-group dynamics shifted.

### 5.3 Recentered influence function regression results: urban–rural analysis

Table 3 reports the RIF variance regression results disaggregated by urban–rural residence. Models 1 and 2 are estimated for the urban sample, and Models 3 and 4 for the rural sample. Within each pair, the odd-numbered models do not control for education, while the even-numbered models further incorporate educational attainment dummies.

**Table 3:** RIF regression results of happiness variance of the urban–rural differences.

	Model 1	Model 2	Model 3	Model 4
	Happiness, variance, urban	Happiness, variance, urban	Happiness, variance, rural	Happiness, variance, rural
Year dummy (Reference group: 2014)				
Year of 2018	−0.073 (0.078)	0.114 (0.078)	−0.075 (0.085)	0.124 (0.087)
Year of 2020	−0.412^***^ (0.080)	−0.126 (0.080)	−0.368^***^ (0.092)	−0.082 (0.093)
Year of 2022	−0.461^***^ (0.081)	−0.078 (0.082)	−0.375^***^ (0.095)	0.024 (0.097)
Education level	No	Yes	No	Yes
Age	0.035^***^ (0.002)	0.009^***^ (0.002)	0.044^***^ (0.002)	0.015^***^ (0.002)
Marriage dummy	−1.008^***^ (0.086)	−0.898^***^ (0.086)	−0.896^***^ (0.098)	−0.937^***^ (0.101)
Female	0.058 (0.057)	−0.105^*^ (0.057)	0.282^***^ (0.065)	−0.086 (0.068)
Province dummy	Yes	Yes	Yes	Yes
_cons	3.250^***^ (0.249)	6.169^***^ (0.287)	2.655^***^ (0.540)	5.717^***^ (0.551)
*N*	52,010	51,452	50,528	49,334
adj. *R*^2^	0.014	0.027	0.016	0.028

Robust Standard errors in parentheses. ^*^p < 0.1, ^**^p < 0.05, ^***^p < 0.01.

For the urban sample, without controlling for education, the coefficients for 2020 and 2022 are −0.412 and −0.461, respectively, both significant at the 1% level. After controlling for education, these coefficients drop to −0.126 (*p* = 0.116) and −0.078 (*p* = 0.339), respectively, and both become statistically insignificant. This attenuation pattern is consistent with the full-sample results: the reduction in happiness variance among urban residents is also driven by the convergence of between-education-group gaps. In addition, the coefficient for 2018 shifts from negative to positive after controlling for education (from −0.073 to 0.114), consistent with the within-group widening trend observed in the full sample.

For the rural sample, without controlling for education, the coefficients for 2020 and 2022 are −0.368 and −0.375, respectively, both significant at the 1% level. After controlling for education, the coefficient for 2020 drops to −0.082 and becomes insignificant (*p* = 0.381), while the coefficient for 2022 even turns positive (0.024, *p* = 0.802). This indicates that the equalization of happiness in rural areas is likewise explained by structural changes along the education dimension.

Taken together, the urban–rural subsample results lead to the following conclusion: the equalization of happiness during the pandemic is manifested as the narrowing of between-education-group gaps in both urban and rural sectors. Regardless of urban–rural residence, once educational attainment is controlled for, the year effects largely disappear.

### 5.4 A period-by-period Oaxaca-Blinder RIF decomposition of happiness variance

Table 4 reports the results of the Oaxaca-Blinder RIF decomposition, revealing three distinct stages in the evolution of happiness variance before and after the pandemic. Pre-pandemic period (2014–2018): The total variance did not change significantly (difference = 0.076, *p* > 0.1). However, the composition effect was significantly negative (−0.055, *p* < 0.01), while the structure effect was significantly positive (0.131, *p* < 0.05). This indicates that the narrowing of between-education-group gaps was already exerting downward pressure on overall variance, but this force was fully offset by a concurrent widening of within-group dispersion. This explains why the year coefficient for 2018 was insignificant in the main regression.

**Table 4:** Oaxaca-Blinder RIF-decomposition of happiness variance difference between (estimating standard RIF-Oaxaca using RIF: var).

	Model 1	Model 2	Model 3
	2014 vs. 2018	2018 vs. 2020	2020 vs. 2022
Earlier year (Group 1)	4.683^***^ (0.042)	4.310^***^ (0.046)	4.269^***^ (0.047)
Later year (Group 2)	4.608^***^ (0.042)	4.683^***^ (0.042)	4.310^***^ (0.046)
Difference	0.076 (0.059)	−0.373^***^ (0.062)	−0.041 (0.065)
Explained (composition effect)	−0.055^***^ (0.016)	−0.147^***^ (0.015)	−0.113^***^ (0.015)
Unexplained (structure effect)	0.131^**^ (0.060)	−0.226^***^ (0.062)	0.072 (0.065)
Explained			
Age	0.014^***^ (0.005)	−0.022^***^ (0.005)	−0.001 (0.003)
Marriage	0.011^***^ (0.004)	0.013^***^ (0.004)	0.018^***^ (0.005)
Female	−0.001 (0.001)	0.001 (0.001)	0.001 (0.001)
Urban	−0.002 (0.002)	−0.003^*^ (0.002)	−0.004^**^ (0.002)
*Edu* (Reference group: Illiterate / Semi-literate)			
Primary school	0.026^***^ (0.005)	0.005 (0.003)	0.015^***^ (0.004)
Middle school	0.013^*^ (0.007)	−0.032^***^ (0.008)	−0.007 (0.007)
High school / vocational school	−0.037^***^ (0.008)	−0.018^**^ (0.008)	−0.019^**^ (0.009)
Junior college / University	−0.101^***^ (0.010)	−0.087^***^ (0.011)	−0.105^***^ (0.013)
Postgraduate	−0.005^***^ (0.002)	−0.007^***^ (0.002)	−0.011^***^ (0.004)
Unexplained			
Age	−0.021 (0.192)	0.387^*^ (0.212)	−0.026 (0.221)
Marriage	−0.370^***^ (0.120)	0.061 (0.123)	−0.001 (0.127)
Female	−0.089 (0.059)	−0.003 (0.063)	−0.018 (0.066)
Urban	0.090 (0.063)	−0.054 (0.068)	0.006 (0.073)
*Edu* (Reference group: Illiterate / Semi-literate)			
Primary school	0.085^**^ (0.042)	−0.043 (0.039)	0.015 (0.043)
Middle school	−0.059 (0.056)	−0.025 (0.059)	0.068 (0.069)
High school / vocational school	−0.103^***^ (0.032)	0.050 (0.037)	−0.012 (0.043)
Junior college / University	−0.040^*^ (0.021)	0.022 (0.029)	−0.010 (0.039)
Postgraduate	−0.001 (0.003)	0.003 (0.003)	−0.008 (0.005)
Province dummy	Yes	Yes	Yes
_cons	1.555^**^ (0.717)	−1.714^**^ (0.786)	0.769 (0.779)

Standard errors in parentheses. ^*^p < 0.1, ^**^p < 0.05, ^***^p < 0.01.

Pandemic shock period (2018–2020): The total variance declined sharply and significantly (difference = −0.373, *p* < 0.01). The composition effect expanded to −0.147, and the structure effect reversed sign to become significantly negative (−0.226, *p* < 0.01). This suggests that between-group convergence and within-group compression began to work in the same direction, jointly driving the equalization of happiness during the pandemic. Pandemic persistence period (2020–2022): The trend toward variance reduction stabilized (difference = −0.041, *p* > 0.1). The composition effect remained significantly negative (−0.113, *p* < 0.01), but the structure effect became insignificant (0.072, *p* > 0.1).

The detailed decomposition further reveals that educational attainment is the dominant driver of the composition effect. The aggregate contributions of the education variables across the three periods are −0.104, −0.139, and −0.127, respectively. Among these, the contribution of university-level education is the most prominent. In contrast, the contributions of age structure, marital status, gender, and urban–rural residence are considerably smaller.

Taken together, these decomposition results confirm that the reduction in happiness variance is attributable to the convergence of mean happiness across educational groups. Specifically, the happiness of the lowest education group rose steadily, while that of the highest education group declined markedly, with the two group means nearly converging by 2022.

## 6 Robustness tests

### 6.1 Alternative distributional measures

To ensure that the core findings are not dependent on a specific distributional statistic, we re-estimated the RIF regressions using the interquartile range (IQR) and the Gini coefficient in place of the variance. Table 5 reports the results of these robustness checks.

**Table 5:** RIF regression results using alternative distributional measures (IQR and Gini).

	Model 1	Model 2	Model 3	Model 4
	IQR	IQR	Gini	Gini
Year dummy (Reference group: 2014)				
Year of 2018	−0.083^***^ (0.025)	0.003 (0.025)	−0.001 (0.001)	0.003^**^ (0.001)
Year of 2020	−0.255^***^ (0.027)	−0.122^***^ (0.028)	−0.006^***^ (0.001)	−0.000 (0.001)
Year of 2022	−0.319^***^ (0.028)	−0.132^***^ (0.029)	−0.005^***^ (0.001)	0.003^**^ (0.001)
Education level	No	Yes	No	Yes
Age	0.018^***^ (0.001)	0.005^***^ (0.001)	0.001^***^ (0.000)	0.000^***^ (0.000)
Marriage dummy	−0.099^***^ (0.024)	−0.085^***^ (0.025)	−0.022^***^ (0.001)	−0.022^***^ (0.001)
Female	0.124^***^ (0.019)	0.003 (0.020)	0.002^**^ (0.001)	−0.003^***^ (0.001)
Urban	−0.295^***^ (0.020)	−0.061^***^ (0.021)	−0.015^***^ (0.001)	−0.006^***^ (0.001)
Province dummy	Yes	Yes	Yes	Yes
_cons	2.743^***^ (0.111)	4.128^***^ (0.116)	0.133^***^ (0.005)	0.189^***^ (0.005)
*N*	102,538	100,786	102,538	100,786
adj. *R*^2^	0.020	0.036	0.023	0.035

Robust Standard errors in parentheses. ^*^p < 0.1, ^**^p < 0.05, ^***^p < 0.01.

For the IQR, the 2018 coefficient drops from −0.083 (*p* < 0.01) to 0.003 and becomes insignificant after controlling for education, while the 2022 coefficient falls from −0.319 (*p* < 0.01) to −0.132 (*p* < 0.01). The contrast with the variance results is instructive. Between 2014 and 2018, the overall variance did not change significantly, whereas the IQR registered a statistically significant decline. This divergence arises because the variance, which is sensitive to extreme values, was pulled in opposite directions—compressed by between-group convergence but inflated by a widening of dispersion at the extremes. The IQR, by excluding the tails of the distribution, isolates the net compression effect of between-group convergence on the middle of the distribution, and thus captures a significant reduction that the variance alone would obscure. After the onset of the pandemic, the decline in the IQR widened substantially: the coefficient was −0.083 in 2018, and reached −0.255 and −0.319 in 2020 and 2022, respectively, and in each case the coefficient fell sharply after controlling for education. This is consistent with the variance results.

For the Gini coefficient, the coefficients for both 2018 and 2022 reverse sign from negative to positive after controlling for education (0.003 and 0.003, respectively, *p* < 0.05). This implies that, once the convergence of mean happiness across educational groups is stripped out, overall happiness inequality was on an upward trajectory both before and during the pandemic. Educational convergence thus served as an important driver of equalization during the pandemic.

Taken together, the results across all three measures confirm the robustness of our core finding: the equalization of happiness during the pandemic stemmed from the convergence of mean happiness across educational groups.

### 6.2 RIF variance regression excluding extreme happiness scores (0 and 10)

Table 6 reports the RIF variance regression results after excluding observations with happiness scores of 0 and 10. Overall, the core findings remain robust after excluding extreme values, though two noteworthy differences emerge.

**Table 6:** RIF variance regression results excluding extreme happiness scores (0 and 10).

	(1)	(2)
	Model 1: Happiness, variance	Model 2: Happiness, variance
Year dummy (Reference group: 2014)		
Year of 2018	−0.191^***^ (0.042)	−0.096^**^ (0.043)
Year of 2020	−0.355^***^ (0.044)	−0.218^***^ (0.044)
Year of 2022	−0.411^***^ (0.044)	−0.228^***^ (0.045)
Education level	No	Yes
Age	0.020^***^ (0.001)	0.008^***^ (0.001)
Marriage dummy	−0.538^***^ (0.045)	−0.516^***^ (0.045)
Female	0.127^***^ (0.031)	0.004 (0.031)
Urban	−0.271^***^ (0.033)	−0.073^**^ (0.034)
Province dummy	Yes	Yes
_cons	2.589^***^ (0.159)	3.940^***^ (0.175)
*N*	77,312	76,060
adj. *R*^2^	0.014	0.020

Robust Standard errors in parentheses. ^*^p < 0.1, ^**^p < 0.05, ^***^p < 0.01.

First, the year coefficients remain significant after controlling for education. In the full sample, the 2022 coefficient falls from −0.433 to −0.045 and becomes insignificant after controlling for education; after excluding extreme values, the 2022 coefficient drops from −0.411 to −0.228 (*p* < 0.01), an attenuation of roughly 45%. This difference indicates that, even after excluding extreme values, between-group educational convergence remains an important driving force behind the reduction in happiness variance. Importantly, the gradient in magnitude between pre- and post-pandemic periods remains robust: the 2018 coefficient without controlling for education is −0.191, compared with −0.355 for 2020 and −0.411 for 2022, consistent with the full-sample finding that happiness variance declined substantially after the onset of the pandemic.

Second, the 2018 coefficient is already significantly negative without controlling for education (−0.191, *p* < 0.01), whereas in the full sample it is −0.077 and insignificant. This change aligns with the IQR results: once the interference of extreme values is removed, the net compression effect of between-group convergence on happiness variance is already discernible before the pandemic. However, in both the trimmed and full samples, the post-pandemic decline in variance far exceeds the pre-pandemic decline, with the coefficients for 2020 and 2022 after controlling for education (−0.218 and −0.228) markedly higher than that for 2018 (−0.096). The direction of the core findings is unaffected by the sample adjustment.

Taken together, the post-pandemic decline in variance consistently exceeds the pre-pandemic decline, and the convergence of mean happiness across educational groups remains an important force driving equalization—a core finding that is not undermined by the exclusion of extreme values.

### 6.3 RIF regression results with panel weights

Table 7 reports the RIF regression results weighted by individual panel weights. The variance results (Models 1–2) show that, without controlling for education, the 2020 and 2022 coefficients are −0.312 (*p* < 0.05) and −0.363 (*p* < 0.01), respectively, indicating a significant post-pandemic decline in variance. After controlling for education, they rise to −0.038 and 0.007, respectively, and both become insignificant, confirming that the convergence of mean happiness across educational groups is a key structural source of the reduction in variance. The Gini coefficient results (Models 3–4) further reinforce this conclusion. Both the 2018 and 2022 coefficients turn significantly positive after controlling for education (0.007 and 0.006, *p* < 0.01 and *p* < 0.05): once the between-group convergence effect is stripped out, happiness inequality exhibits an upward trend both before and during the pandemic. Overall, the weighted and unweighted results are consistent, confirming that the core findings are robust and not driven by sample composition.

**Table 7:** RIF regression results using panel weights.

	Model 1	Model 2	Model 3	Model 4
	Variance	Variance	Gini	Gini
Year dummy (Reference group: 2014)				
Year of 2018	0.045 (0.125)	0.237^*^ (0.124)	0.003 (0.003)	0.007^***^ (0.003)
Year of 2020	−0.312^**^ (0.127)	−0.038 (0.127)	−0.003 (0.003)	0.003 (0.003)
Year of 2022	−0.363^***^ (0.113)	0.007 (0.114)	−0.001 (0.002)	0.006^**^ (0.002)
Education level	No	Yes	No	Yes
Age	0.043^***^ (0.003)	0.014^***^ (0.004)	0.001^***^ (0.000)	0.000^***^ (0.000)
Marriage	−1.199^***^ (0.159)	−1.136^***^ (0.163)	−0.027^***^ (0.003)	−0.026^***^ (0.003)
Female	0.125 (0.089)	−0.142 (0.091)	0.002 (0.002)	−0.003^*^ (0.002)
Urban	−0.726^***^ (0.085)	−0.254^***^ (0.090)	−0.016^***^ (0.002)	−0.007^***^ (0.002)
_cons	3.100^***^ (0.325)	6.353^***^ (0.368)	0.120^***^ (0.007)	0.182^***^ (0.008)
*N*	53,287	52,769	53,287	52,769
adj. *R*^2^	0.020	0.035	0.023	0.036

Robust Standard errors in parentheses. ^*^p < 0.1, ^**^ *p* < 0.05, ^***^ *p* < 0.01.

## 7 Discussion

The decline in happiness inequality observed in this study adds to the emerging literature on the distributional consequences of COVID-19 for subjective well-being. A large body of international evidence documents that the pandemic disproportionately harmed lower-income and lower-educated groups in terms of both health and economic outcomes, yet its implications for happiness inequality appear more context-dependent. Prior research in Japan has similarly reported a narrowing of happiness gaps during the pandemic, suggesting that the equalization pattern observed in China may not be unique.

Several features of the Chinese context may help account for these findings. First, the government-led allocation of essential supplies and the rapid expansion of social assistance provided a crucial safety net for the most vulnerable groups. This government-led buffer prevented a further deterioration of happiness at the lower end of the distribution, thereby compressing the overall variance.

It is worth noting that the convergence of mean happiness across educational groups was already underway before the pandemic. This trend is unlikely to be an isolated statistical artifact; rather, it may be rooted in a series of policies implemented since the 18th National Congress of the Communist Party of China in 2012 aimed at promoting social equity and equalizing public services. Over the past decade, large-scale anti-poverty programs, the revitalization of rural areas, the integration of urban and rural resident medical insurance, and the balanced development of compulsory education have substantially improved living conditions and social protection for rural and less-educated populations. At the same time, the rapid expansion of higher education has raised the aspirations of the highly educated, while intensifying competition in the job market may have eroded their relative advantage in subjective well-being. The cumulative effect of these policies provides a longer-term structural backdrop for the equalization of happiness documented in this paper. In this sense, the pandemic shock may have accelerated and amplified an underlying trend already set in motion by earlier policy orientations: the strengthened government safety net prevented a further deterioration of happiness at the lower end. Our empirical findings can thus be read not only as an assessment of the distributional consequences of pandemic-era policy responses, but also as an indirect reflection of the broader trajectory of China’s social policy over the longer term.

Our analysis does not directly test the mechanisms proposed above; the role of zero-COVID policies, social assistance, and psychological adaptation remain suggestive interpretations that warrant further research. Whether the narrowing of happiness gaps persists after the abandonment of the zero-COVID policy in late 2022 remains an open empirical question.

## 8 Limitations

Several limitations of this study should be acknowledged. First, the identification strategy used in this paper relies on a before-after comparison of happiness inequality across survey waves, and therefore does not constitute a credible causal design in the strict sense. The observed decline in happiness variance between the pre-pandemic and pandemic periods coincides with the onset of COVID-19, but may also reflect other concurrent trends or shocks—including macroeconomic fluctuations, policy reforms, and demographic shifts—that are not separately identified in our framework. Our analysis mitigates this concern in several ways. By incorporating the earliest available survey wave, we extend the pre-pandemic observation window, allowing us to examine the trajectory of happiness inequality over a longer period prior to COVID-19. The descriptive evidence shows that happiness variance remained relatively stable across the pre-pandemic waves, with no statistically significant differences between them. The RIF regression further confirms that the pre-pandemic year coefficient is statistically insignificant, while the period-by-period Oaxaca-Blinder decomposition reveals that the negligible total change in the pre-pandemic period masks two offsetting forces—between-group convergence and within-group widening—neither of which produced a net reduction in variance. These patterns are inconsistent with a strong pre-existing downward trend, lending support to the interpretation that the pandemic played an important role in the subsequent decline. Nevertheless, the association documented here should still be interpreted as suggestive rather than definitively causal.

Second, the convergence of mean happiness across educational groups—identified in our analysis as the primary mechanism—was already underway before the pandemic, as evidenced by the persistent narrowing of the happiness gap between the highest and lowest education groups throughout the pre-pandemic period. This raises the possibility that at least part of the equalization we observe would have occurred even in the absence of COVID-19. The precise contribution of the pandemic relative to the pre-existing trend, however, remains difficult to quantify.

Third, a further limitation is that our regression sample contains only two pre-pandemic waves (2014 and 2018). With only two pre-pandemic time points, the stability of pre-COVID trends cannot be formally tested. While the descriptive evidence and period-by-period decomposition provide suggestive support for trend stability, a longer pre-pandemic time series would be required to more rigorously validate this assumption.

## 9 Conclusion

This paper examines the evolution of happiness inequality in China before and after the COVID-19 pandemic using CFPS panel data from 2014 to 2022. Employing RIF variance regression and Oaxaca-Blinder RIF decomposition, we find that the variance of happiness declined significantly after the onset of the pandemic, with the 2020 and 2022 coefficients being substantially larger than the pre-pandemic change. This reduction was driven by the convergence of mean happiness across educational groups—a trend already evident before the pandemic—with the happiness of the least educated rising steadily, while that of the most educated declined markedly, narrowing the between-group gap from 0.743 in 2014 to merely 0.025 in 2022. This process was reinforced by the narrowing of within-group dispersion for most educational groups in the early pandemic period (2018–2020). Once educational attainment is controlled for, the year coefficients attenuate substantially. The Oaxaca-Blinder decomposition further reveals that the educational convergence underlying the composition effect was already underway before the pandemic. These findings are robust to alternative distributional measures (IQR and Gini coefficient), the exclusion of extreme happiness scores, the use of panel weights, and urban–rural subsample analysis. Whether this equalization persists beyond the zero-COVID period remains an open question for future research.

## Data Availability

Publicly available datasets were analyzed in this study. This data can be found at: http://www.isss.pku.edu.cn/cfps/gycfps/cfpsjj/index.htm.
